# Supplementary figures and images for: The ACF chromatin-remodeling complex is essential for Polycomb repression
Source: eLife. 2022 Mar 8;11:e77595. doi: 10.7554/eLife.77595 (PMC9038196; doi:10.7554/eLife.77595)

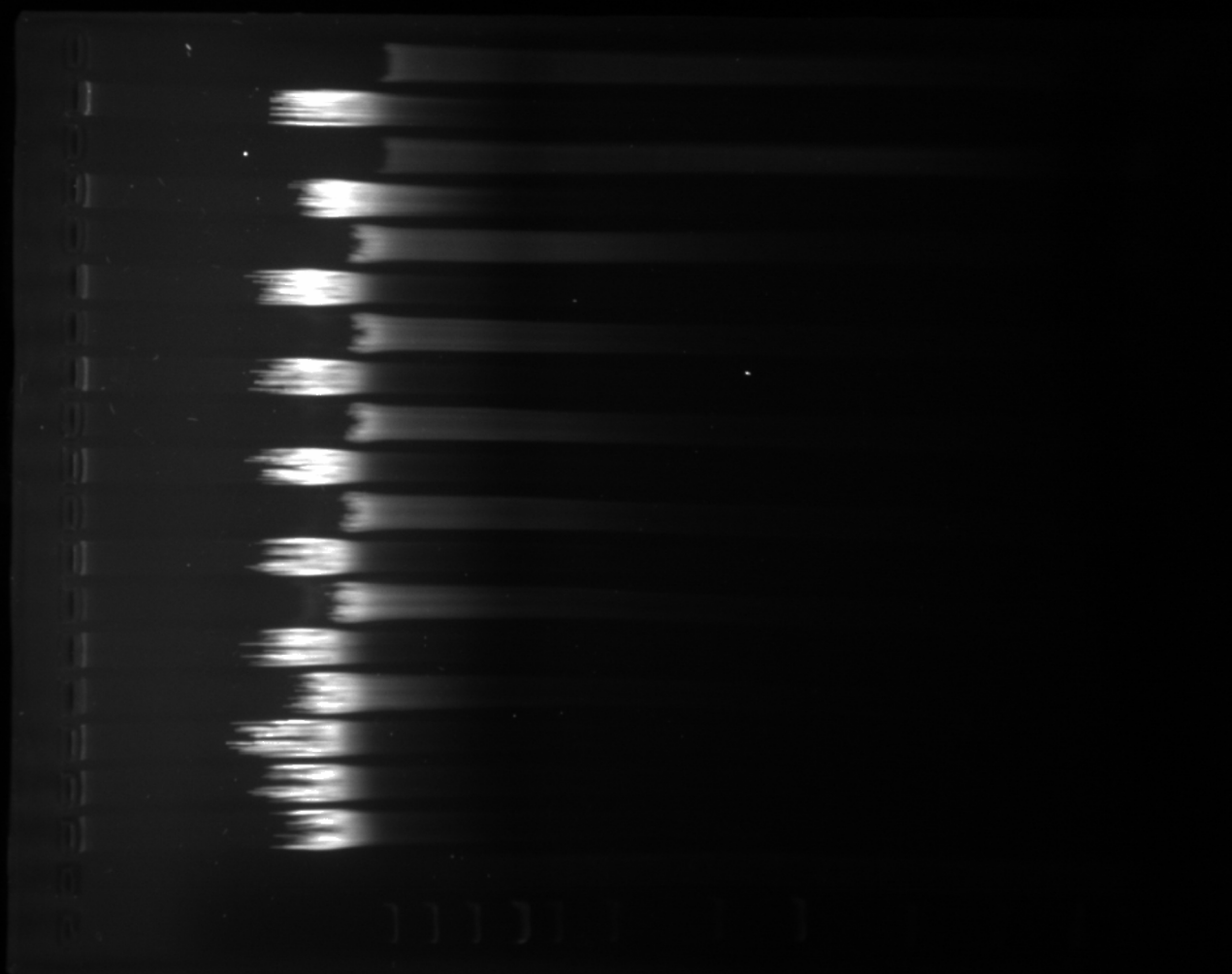

Supplement: Figure 4—figure supplement 1—source data 1. [file elife-77595-fig4-figsupp1-data1.zip › Figure4_FigureSupplement1_SourceData1.pdf]

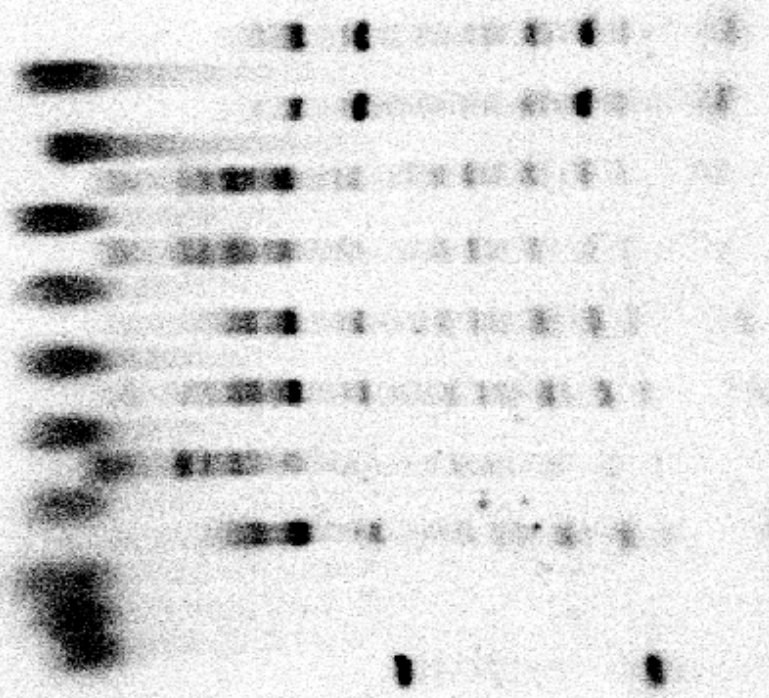

Supplement: Figure 4—figure supplement 1—source data 2. [file elife-77595-fig4-figsupp1-data2.pdf]

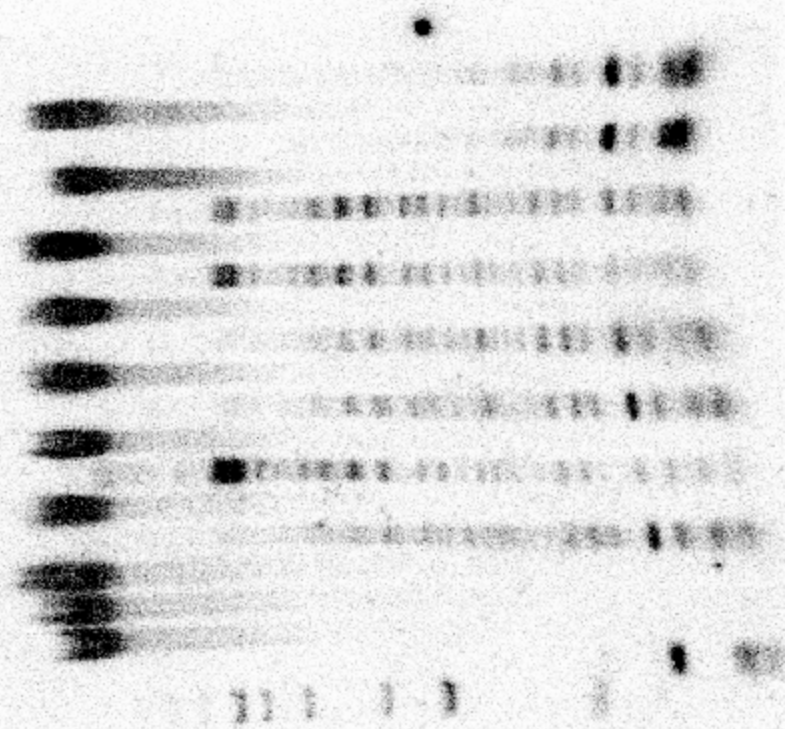

Supplement: Figure 4—figure supplement 1—source data 3. [file elife-77595-fig4-figsupp1-data3.pdf]

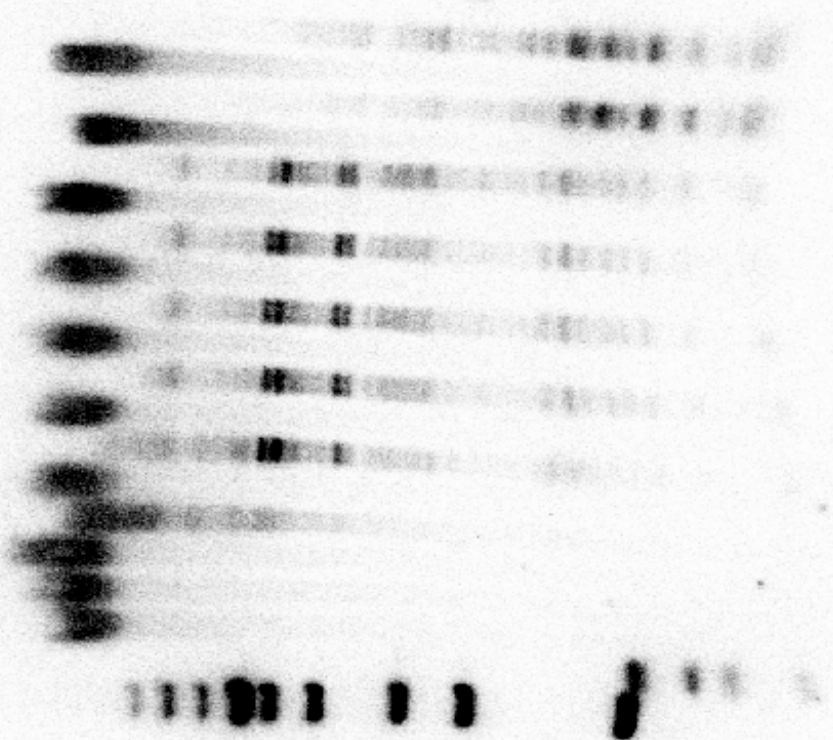

Supplement: Figure 4—figure supplement 1—source data 4. [file elife-77595-fig4-figsupp1-data4.pdf]

EPR-1-

Dam

ACF1-Dam

WT

$\Delta eed$

$\Delta set-7$

unrelated

|| - | - | - | - | - | - | - |

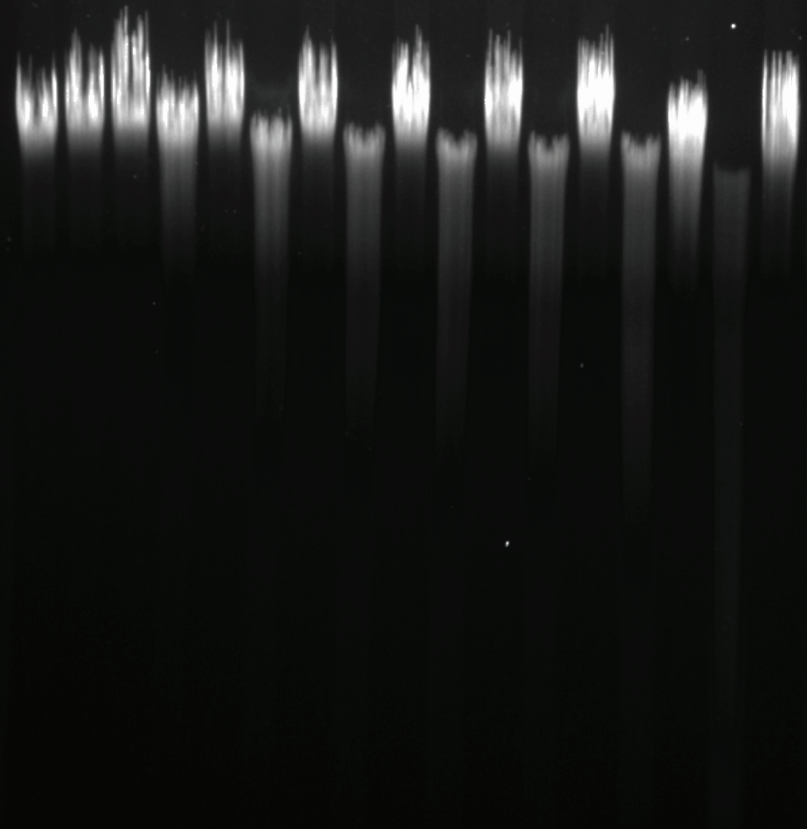

Et-Br

Supplement: Figure 4—figure supplement 1—source data 5. [file elife-77595-fig4-figsupp1-data5.pdf]

|    |   | EPR-1-Dam    |   |   | ACF1-Dam       |   |   |           |  |
|----|---|--------------|---|---|----------------|---|---|-----------|--|
| WT |   | $\Delta eed$ |   |   | $\Delta set-7$ |   |   | unrelated |  |
|    | - | -            | - | - | -              | - | - | -         |  |

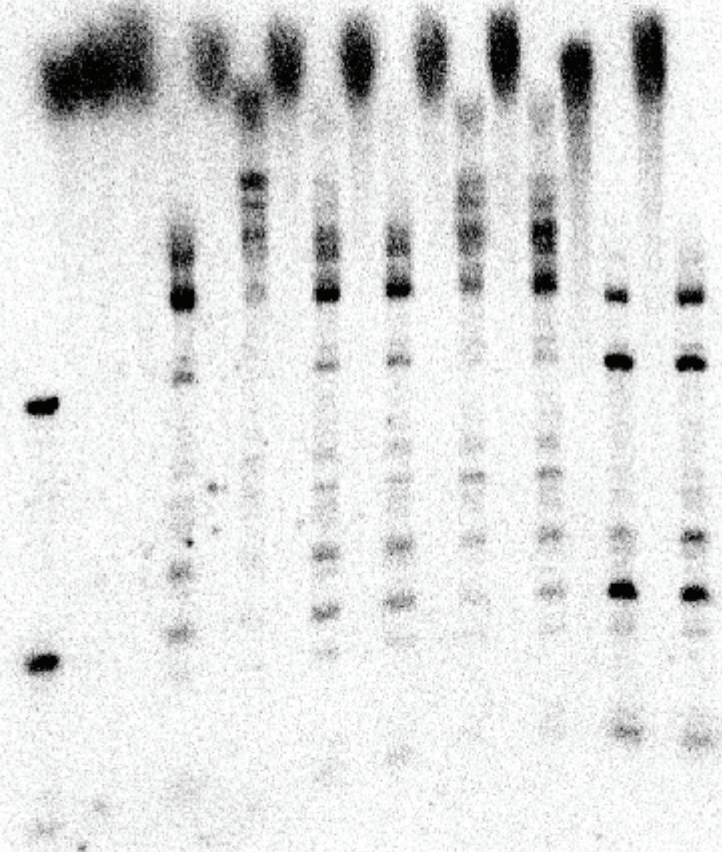

NCU05173

Supplement: Figure 4—figure supplement 1—source data 6. [file elife-77595-fig4-figsupp1-data6.pdf]

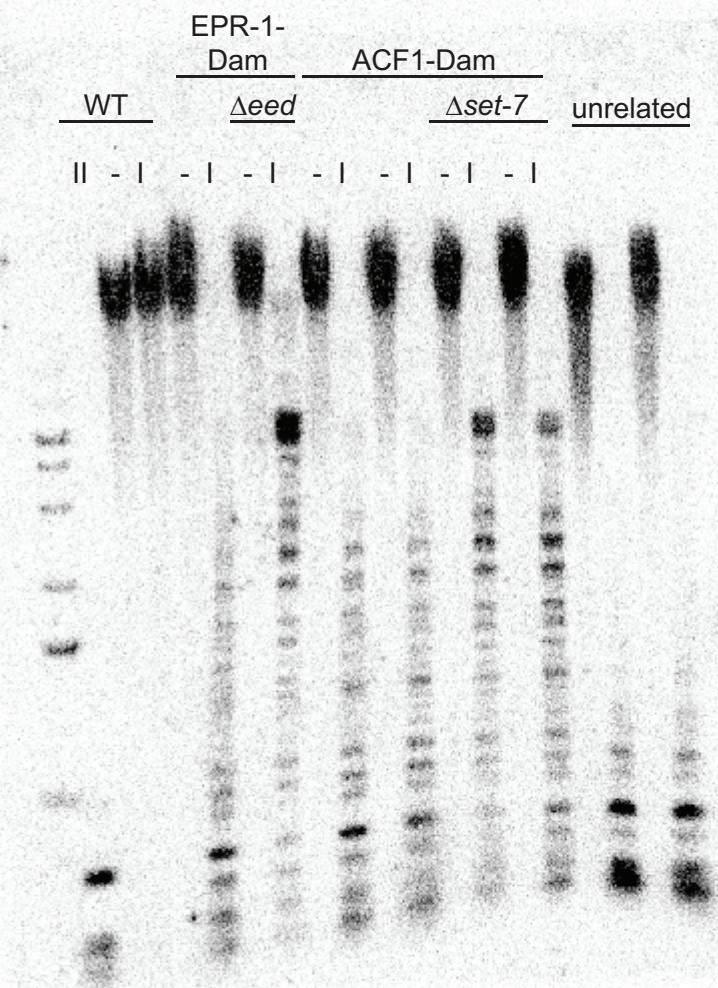

Tel VIII

Supplement: Figure 4—figure supplement 1—source data 7. [file elife-77595-fig4-figsupp1-data7.pdf]

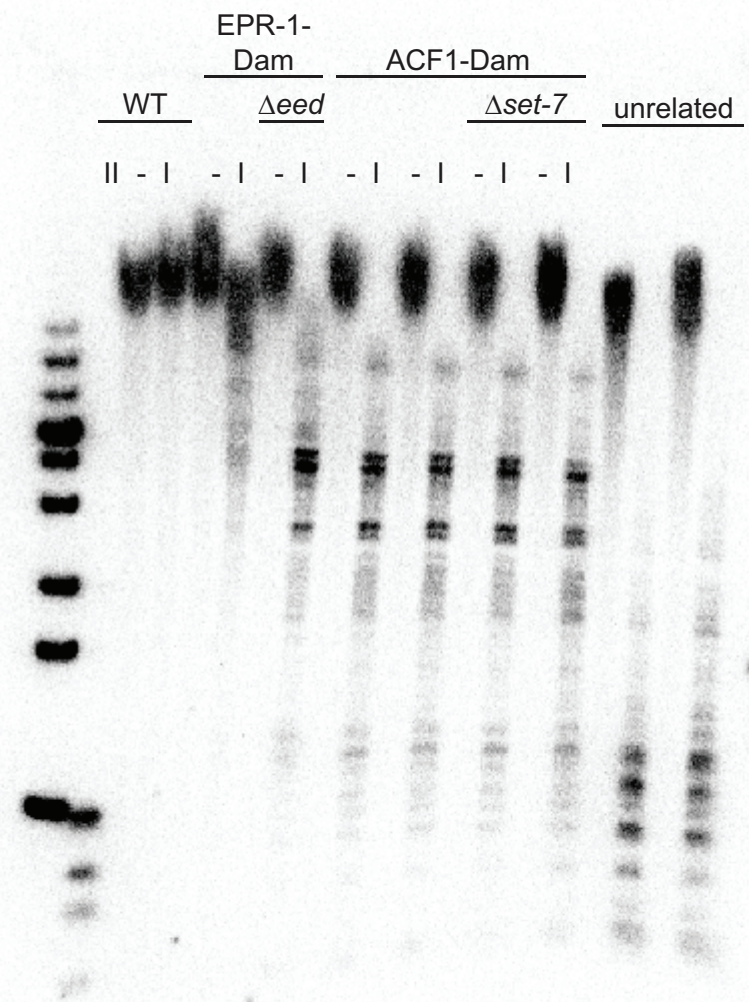

*his-3*

Supplement: Figure 4—figure supplement 1—source data 8. [file elife-77595-fig4-figsupp1-data8.pdf]
